# The role of anxiety and depression in suicidal thoughts for autistic and non‐autistic people: A theory‐driven network analysis

**DOI:** 10.1111/sltb.12954

**Published:** 2023-03-28

**Authors:** Mirabel K. Pelton, Hayley Crawford, Kim Bul, Ashley E. Robertson, Jon Adams, Derek de Beurs, Jacqui Rodgers, Simon Baron‐Cohen, Sarah Cassidy

**Affiliations:** ^1^ Institute for Health and Wellbeing, Centre for Intelligent Healthcare Coventry University Coventry UK; ^2^ Mental Health and Wellbeing Unit, Warwick Medical School University of Warwick Coventry UK; ^3^ School of Psychology & Neuroscience University of Glasgow, University Avenue Glasgow UK; ^4^ Autistic Advocate and Researcher Portsmouth UK; ^5^ Trimbos Instituut Utrecht The Netherlands; ^6^ Population Health Sciences Institute Sir James Spence Institute, Newcastle University, Royal Victoria Infirmary Newcastle UK; ^7^ Autism Research Centre, Department of Psychiatry University of Cambridge Cambridge UK; ^8^ School of Psychology University of Nottingham, University Park Nottingham UK

**Keywords:** autism, interpersonal theory of suicide, network analysis

## Abstract

**Background:**

Autistic adults experience more frequent suicidal thoughts and mental health difficulties than non‐autistic adults, but research has yet to explain how these experiences are connected. This study explored how anxiety and depression contribute to suicidal thoughts according to the Interpersonal Theory of Suicide for autistic and non‐autistic adults.

**Methods:**

Participants (autistic adults *n* = 463, 61% female; non‐autistic *n* = 342, 64% female) completed online measures of anxiety, depression, thwarted belonging, and perceived burdensomeness. Network analysis explored whether: (i) being autistic is a risk marker for suicide; and (ii) pathways to suicidal thoughts are consistent for autistic and non‐autistic adults.

**Results:**

Being autistic connected closely with feeling like an outsider, anxiety, and movement, which connected to suicidal thoughts through somatic experiences, low mood, and burdensomeness. Networks were largely consistent for autistic and non‐autistic people, but connections from mood symptoms to somatic and thwarted belonging experiences were absent for autistic adults.

**Conclusion:**

Autistic people experience more life stressors than non‐autistic people leading to reduced coping, low mood, and suicidal thoughts. Promoting belonging, reducing anxiety, and understanding the role of movement could inform suicide prevention for autistic people. Research should accurately capture autistic lived experience when modeling suicide to ensure suicide prevention meets autistic needs.

## INTRODUCTION

Suicide accounts for the deaths of over 700,000 people each year (World Health Organization, 2021) and autistic people^1^—recently estimated to number at least 80 million worldwide (Lord et al., 2022)—are now recognized as over‐represented in those figures (Cassidy et al., 2022; Hirvikoski et al., 2016; Kirby et al., 2019; Kõlves et al., 2021). Autism is diagnosed by the observable presence of social communication, sensory differences, and restricted interests (American Psychiatric Association, 2013) and a recent UK study reported evidence of autism or possible autism in around 41% of those who died by suicide (Cassidy et al., 2022). Thus, there is an urgent need to understand and provide evidence‐based interventions for autistic people to meet global suicide prevention targets (Cassidy, Cogger‐Ward, et al., 2021; Cassidy, Robertson, et al., 2020). One limiting factor in guiding interventions is the absence of suicide theory and models that accurately describe the experiences of autistic people (Cassidy, Cogger‐Ward, et al., 2021). Our earlier research reported the Interpersonal Theory of Suicide (ITS) may be relevant for autistic adults (Pelton & Cassidy, 2017), but the model explains only one‐third of the variance in lifetime suicidal thoughts and behaviors for autistic adults compared with non‐autistic adults (Pelton et al., 2020b). One possibility is that mental health difficulties, such as anxiety and depression, could have a greater influence on suicidal thoughts and behaviors for autistic than non‐autistic adults. Mental health difficulties, such as anxiety and depression are reported by up to 80% of autistic adults (Lever & Geurts, 2016) and have been significantly associated with suicidal thoughts, behaviors, and death by suicide for autistic young people and adults (Jokiranta‐Olkoniemi et al., 2021; Kõlves et al., 2021; Zahid & Upthegrove, 2017), but we do not yet know *how* these experiences are connected. Reducing persistent distress caused by suicidal thoughts has been identified as a clinical priority for autistic adults (South et al., 2020) that could reduce future death by suicide (Large et al., 2021). Thus, in this study, we set out to explore the role of anxiety and depression in the development of suicidal desire according to the ITS and whether this differs for autistic and non‐autistic adults.

**FIGURE 1:**
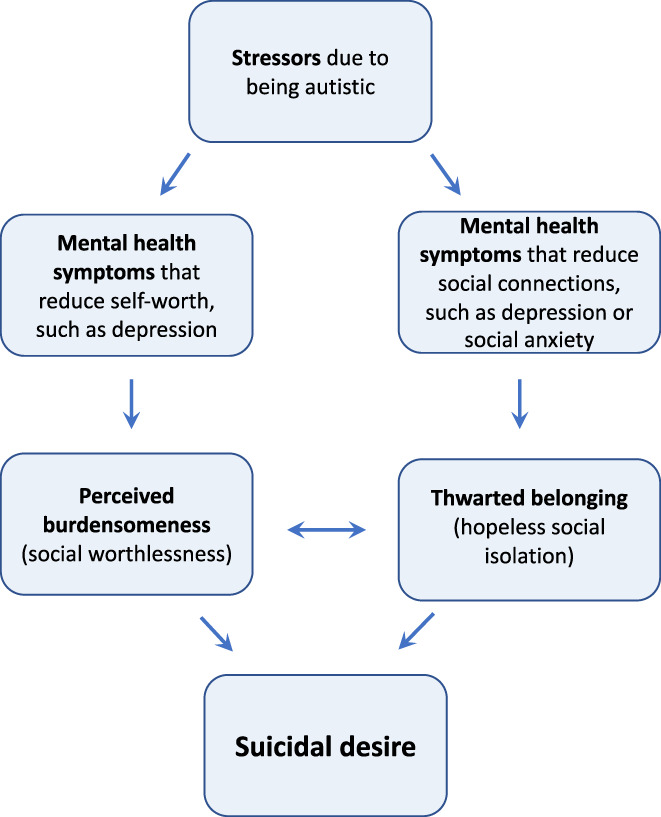
Hypothesized association between mental health difficulties, interpersonal theory of suicide proximal risk markers and suicidal thoughts showing possible influence of minority stress for autistic people.

According to the ITS, in any population group, suicidal desire develops from the interaction of perceived burdensomeness (social worthlessness) with thwarted belonging (hopeless social isolation) (Joiner, 2005; Van Orden et al., 2010). As shown in Figure 1, mental health symptoms hypothetically contribute to suicidal desire because they increase the experience of these proximal risk markers (Davidson et al., 2011; Kleiman et al., 2014; Silva et al., 2015). Testing these pathways for autistic adults, however, requires a transdiagnostic approach to modeling mental health (Lombardo et al., 2019; Weiss, 2014). Higher rates of misdiagnosed and co‐occurring mental health difficulties (Camm‐Crosbie et al., 2019) are attributed to differences between autistic and non‐autistic people in the way that mental health—such as depression (Cassidy, Bradley, Bowen, et al., 2018; Cassidy, Bradley, Cogger‐Ward, et al., 2021), anxiety (Boulter et al., 2014; Rodgers et al., 2020; Rodgers & Ofield, 2018), thwarted belonging, and perceived burdensomeness (Pelton et al., 2020a)—are conceptualized and measured so comparisons based on scale total scores may not be accurate. One alternative is the network approach, which conceptualizes mental health difficulties as the interaction of their individual symptoms in contrast with the *common cause* or *medical model* (Borsboom, 2017; Borsboom & Cramer, 2013; Borsboom et al., 2021). Individual symptoms or experiences, such as hopelessness, feeling nervous, or like an outsider are *nodes* within the network while *edges* describe the strength of association between two nodes (Borsboom, 2017). Network analysis is particularly recommended for estimating and visualizing the complexity of suicidal thoughts (De Beurs, 2017) and specifically for (i) generating novel, putative theoretical pathways (Haslbeck et al., 2022) and (ii) understanding differences between patient groups (De Beurs, 2017). Thus, these are the aims of the current study.

One possibility is that being autistic represents a distal risk marker for suicide due to minority stress (Botha & Frost, 2020). As shown in Figure 1, being autistic could lead to multiple life stressors, making it more likely that an individual experiences anxiety and depression (Lever & Geurts, 2016), thwarted belonging, burdensomeness (Pelton et al., 2020b) and suicidal thoughts (Cassidy et al., 2014) than a non‐autistic person. Empirical research supports this: autistic traits are a unique risk marker for suicidal thoughts and behaviors in autistic and non‐autistic samples (Cassidy et al., 2022; Cassidy, Bradley, Shaw, et al., 2018; Pelton & Cassidy, 2017; Richards et al., 2019; Takara & Kondo, 2014; Upthegrove et al., 2018). Simple pathways have been described from (i) autistic traits through perceived burdensomeness and thwarted belonging (Pelton et al., 2020b); (ii) loneliness through depression (Hedley, Uljarević, Foley, et al., 2018); and (iii) social dissatisfaction and loneliness through perceived burdensomeness (Dow et al., 2021); to suicidal thoughts and behaviors for autistic adults. However, these modeling studies have included only simply linear relationships and have employed scale totals using measures designed for non‐autistic adults. Furthermore, network analysis has already extended our understanding of the interaction of these variables for non‐autistic people: perceived burdensomeness and low mood symptoms (such as feeling depressed or hopeless) are reported closely, directly connected to suicidal thoughts while thwarted belonging and anxiety are more distal or less strongly connected (Beard et al., 2016; De Beurs et al., 2019; Fried et al., 2016; Ordóñez‐Carrasco et al., 2021; Schönfelder et al., 2021; Suh et al., 2021), but studies, to date, have not included autistic samples. Thus, this study will use network analysis to visualize and estimate more complex interactions than traditional statistical analytic techniques to understand the role of, for example, restlessness or appetite preferences, without needing to attribute these to anxiety, depression, or autistic characteristics.

Finally, researchers propose that being autistic may moderate connections between risk markers (Lai et al., 2017; Pelton & Cassidy, 2017) and network analysis provides a range of tools to simultaneously test a range of propositions: first, autistic thinking styles, such as perseverative thinking (Arwert & Sizoo, 2020; Lai et al., 2017; South et al., 2020), may make it harder to switch away from negative thoughts and relatively hasten the development of suicidal thoughts compared with non‐autistic people. This is in line with the network approach which argues that connections between risk markers are stronger in vulnerable compared with less vulnerable groups (van Borkulo et al., 2015). Second, differences in emotional experience, such as alexithymia (difficulty identifying and expressing emotional states (Bird & Cook, 2013)) could mean that emotional symptoms are understated, attenuating connections between emotional risk markers, such as low mood or anxiety, with other risk markers (Costa et al., 2020; Pelton & Cassidy, 2017; Pelton et al., 2020b). Third, overlapping autistic characteristics and somatic depression symptoms (such as appetite or sleep differences) (Gotham et al., 2015) could suggest that somatic experiences are more influential in maintaining the symptom network for autistic compared with non‐autistic adults (Montazeri et al., 2020). However, to date, none of these propositions have been tested in a matched sample of autistic and non‐autistic adults. Thus, the current study will undertake exploratory analyses to explore whether and how connections between risk markers differ between autistic and non‐autistic adults.

Thus, the current study will undertake an item‐level network analysis to explore (i) whether and how being autistic represents a distal risk marker for suicide and (ii) whether and how risk markers interact differently for autistic compared with non‐autistic adults. We regard these analyses as exploratory due to an absence of previous research and are open to data‐driven results. Based on previous empirical research we expect to find autistic adults report more frequent experiences of anxiety, depression, thwarted belonging, perceived burdensomeness, and suicidal thoughts. In line with the network approach, there will be multiple interactions within and between items measuring distinct constructs.

## MATERIALS AND METHODS

### Participants and procedure

Data in this study are described elsewhere (Pelton et al., 2020a, 2020b). Participants were 805 complete records retained from an online survey of general population autistic (*n* = 463 [58% sample], 61% female) and non‐autistic (*n* = 342 [42%], 64% female) adults (Table 1). We recruited autistic adults via Cambridge Autism Research Database, West Midlands and UK autism organizations, including Autistica (UK‐based autism research charity), and non‐autistic adults via Cambridge Psychology Database, Coventry University psychology research participation scheme, suicide‐focussed websites, and social media.

**TABLE 1:** Demographic information.

	Non‐autistic (*n* = 342)	Autistic (*n* = 463)	*p*
Age (mean (SD))	41.31 (15.7)	41.55 (13.9)	0.82
AQ‐S total (mean (SD))	60.92 (12.7)	89.44 (12.0)	<0.01
Gender (%)			
Male	118 (34.7)	150 (32.5)	0.01
Female	219 (64.4)	282 (61.0)	
Not male or female	2 (0.6)	28 (6.1)	
Prefer not to say	1 (0.3)	2 (0.4)	
PHQ‐9 total (mean (SD))	7.64 (6.77)	13.81 (7.43)	<0.01
GAD‐7 total (mean (SD))	6.56 (5.39)	12.02 (6.08)	<0.01
INQ‐10 thwarted belonging (mean (SD))	16.55 (8.02)	25.57 (6.77)	<0.01
INQ‐10 burdensomeness (mean (SD))	8.70 (6.04)	14.30 (7.92)	<0.01
In full‐time employment (*n* (%))	144 (42.1)	143 (30.9)	0.01
Highest academic qualification (*n* (%))			
GCSE/O‐Level/NVQ level 1 or 2	17 (5.0)	45 (9.7)	<0.01
A‐Level/Higher/NVQ level 3/BTEC/GNVQ	43 (12.6)	59 (12.8)	
Higher national diploma	11 (3.2)	26 (5.6)	
Undergraduate degree	83 (24.4)	137 (29.7)	
Postgraduate degree	153 (45.0)	156 (33.8)	
No school certificate or any qualifications	5 (1.5)	8 (1.7)	
Other	28 (8.2)	31 (6.7)	
Additional neurodevelopmental condition (*n* (%))			
Yes	28 (8.2)	127 (27.5)	<0.01
No	312 (91.5)	332 (71.9)	
Prefer not to say	1 (0.3)	3 (0.6)	
Dyspraxia (%)	2 (0.6)	40 (8.6)	<0.01
Learning disability (%)	2 (0.6)	13 (2.8)	0.04
Learning difficulty (%)	2 (0.6)	14 (3.0)	0.03
Dyscalculia (%)	2 (0.6)	14 (3.0)	0.03
Dyslexia (%)	9 (2.6)	45 (9.7)	<0.01
Attention‐deficit hyperactivity (%)	14 (4.1)	64 (13.8)	<0.01
Developmental delay (%)	0 (0.0)	8 (1.7)	0.04
Other (%)	2 (0.6)	19 (4.1)	<0.01
Current mental health diagnosis (*n*, (%))			
Yes	102 (30.0)	298 (64.6)	<0.01
No	235 (69.1)	162 (35.1)	
Prefer not to say	3 (0.9)	1 (0.2)	
Depression (%)	76 (22.2)	238 (51.4)	<0.01
Anxiety (%)	73 (21.3)	248 (53.6)	<0.01
OCD (%)	7 (2.0)	46 (9.9)	<0.01
Bipolar (%)	4 (1.2)	30 (6.5)	<0.01
Personality disorder (%)	5 (1.5)	46 (9.9)	<0.01
Schizophrenia (%)	2 (0.6)	7 (1.5)	0.37
Anorexia (%)	3 (0.9)	33 (7.1)	<0.01
Bulimia (%)	1 (0.3)	12 (2.6)	0.02
Epilepsy (%)	9 (2.6)	13 (2.8)	1.0
Chronic fatigue (%)	11 (3.2)	31 (6.7)	0.04
Tourette's (%)	0 (0.0)	8 (1.7)	0.04
Other (%)	10 (2.9)	59 (12.7)	<0.01
Lifetime reported suicidal thoughts and behaviors (*n* (%))			
No past suicidal thoughts/ behaviors	106 (31.7)	24 (5.4)	<0.01
Past suicidal ideation	120 (35.9)	79 (17.7)	
Past suicide plan	73 (21.9)	187 (41.8)	
Past suicide attempt	35 (10.5)	157 (35.1)	

Participants gave informed consent via Qualtrics, were informed about question content in each section, prompted to take breaks, and given information about support services. Autistic adults (one male and one female) reviewed study materials, clarified instructions, advised on questionnaire selection, interpreted results, and developed the model. Coventry University Faculty of Health and Life Sciences Ethics Committee (ethics approval P61841, approved on 12.12.2018) and the Autism Research Centre, University of Cambridge approved the study.

### Measures

Demographics: Participants' self‐reported age, gender, employment status, mental health difficulties, additional neurodevelopmental conditions, and autism diagnosis.

*Thwarted belongingness and perceived burdensomeness* were measured using *The Interpersonal Needs Questionnaire 10* (*INQ‐10*), a 10‐item scale containing thwarted belonging and perceived burdensomeness subscales (Van Orden et al., 2012). We chose the INQ‐10 over the INQ‐15 to avoid frustration from similarly worded questions raised by our design group and given equivalent validity (Hill et al., 2015; Thwarted belonging *α* = 0.93, perceived burdensomeness *α* = 0.91 in this sample).

*Depression* was measured using 9‐item *Patient Health Questionnaire* (*PHQ‐9*) (Kroenke et al., 2001) which asks how frequently depression symptoms are experienced over the past 2 weeks with a four‐item ordinal scale: “not at all” (0), “several days” (1), “more than half the days” (2), and “nearly every day” (3). Item 9 measures thoughts of suicide and self‐harm and has been used here to measure current suicidal thoughts as in previous studies (de la Torre et al., 2021; Penfold et al., 2021; Quinlivan et al., 2017) (*α* = 0.92 in this sample).

*Anxiety* was measured using 7‐item *Generalized Anxiety Disorder* (*GAD‐7*) (Spitzer et al., 2006), which measures cognitive and emotional symptoms of anxiety on the same four‐item ordinal scale as the PHQ‐9. PHQ9 and GAD7 have been designed for non‐autistic population but used in research amongst autistic adults (Griffiths et al., 2019; Vasa & Mazurek, 2015; *α* = 0.92 in this sample).

*Autistic characteristics* were measured using the *Autism Quotient Short Form* (*AQ‐S*). The AQ‐S is a 28‐item subset of the AQ‐50 with a four‐item response scale from 1 “definitely agree” to 4 “definitely disagree” (Hoekstra et al., 2011). The AQ‐S demonstrates the same latent factors in autistic and non‐autistic adults (Murray et al., 2014; *α* = 0.88 non‐autistic, 0.87 autistic in this sample).

*Self‐reported autism diagnosis* shows up to 99.6% concordance with clinical diagnosis in validation studies (Allison et al., 2012; Daniels et al., 2012; Fombonne et al., 2022). Self‐reported diagnosis allows for larger samples for statistical modeling and online participation, which can increase disclosure of sensitive information, as in previous studies (Cassidy, Bradley, et al., 2020; Cassidy, Bradley, Shaw, et al., 2018). In this sample, total scores on the AQ‐S are above/below suggested cut‐off of 65 indicative of autism for autistic (mean = 89.44) and non‐autistic people (mean = 60.92) respectively (shown in Table 1; Hoekstra et al., 2011). We also included self‐diagnosed and possibly (awaiting diagnosis) autistic people because many autistic adults remain undiagnosed due to the historic development of diagnostic criteria (Lai & Baron‐Cohen, 2015; Russell et al., 2022). Bias in diagnostic tests and difficulties accessing services mean autism is less likely to be diagnosed in women and ethnic minority groups and, thus, these may represent high‐risk groups (Constantino et al., 2020; McCrossin, 2022; Roman‐Urrestarazu et al., 2021; Russell et al., 2022; Tromans et al., 2020).

*Lifetime suicidal thoughts and behaviors* are measured using item 1 of the *Suicidal Behaviors Questionnaire* revised (Osman et al., 2001), which demonstrates equivalent measurement properties in autistic and non‐autistic adults (Cassidy, Bradley, et al., 2020) and as in previous studies (Pelton & Cassidy, 2017). Total scores on the SBQ‐R are recommended not to be compared between autistic and non‐autistic people due to non‐invariance of items 3 and 4 (Cassidy, Bradley, et al., 2020).

### Analytic approach

#### Network models or Gaussian graphical models

Gaussian graphical models (GGMs) are novel network estimation techniques for ordinal and continuous variables in between‐subject data. Edges connecting nodes represent partial correlations: the connection strength between two nodes while controlling for all others in the network. See Borsboom et al. (2021) for primer on the network approach. Analyses were conducted in R (v 4.0.5; R Core Team, 2021).

### Summary of the analyses

We explored data distribution and how the data split according to autism diagnosis (autistic, non‐autistic, and possibly autistic) and gender (female, male, not male or female) using the *networktree* package (Jones et al., 2020). Next, to avoid problems of multi‐collinearity between nodes we combined theoretical knowledge with the *goldbricker* function from the *network* *tools* package (Jones, 2020) to identify potential overlapping constructs and combine node scores using principal component analysis, similar to (Barthel et al., 2020; Lass et al., 2020). We estimated two networks using individual items of the INQ‐10, PHQ‐9, GAD‐7: first, in the whole sample network, we used all variables plus the categorical variable *autism* to estimate a network using *mgm* package (Haslbeck & Waldorp, 2020)to explore whether and how being autistic is connected to suicidal thoughts through network items. Second, we split the data and used the *EstimateGroupNetwork* package (Costantini et al., 2019) to estimate networks for autistic and non‐autistic people. We used the *NetworkComparisonTest* (van Borkulo et al., 2017) to test for differences between autistic and non‐autistic networks in (i) overall inter‐connectivity (*“global density”*: total sum of edges in each network); (ii) individual edge weights; and (iii) or relative inter‐connectedness of nodes (termed *centrality estimates*). We use *expected influence* (total sum of edge weights on a given node taking into account negative edge weights) as other centrality estimates are less reliable (Robinaugh et al., 2016). See Appendix S1 for technical details and r script.

## RESULTS

### Descriptive statistics

Table 2 shows mean individual item scores for autistic and non‐autistic people. Ninety‐five percent of autistic people (68% non‐autistic) reported past suicidal thoughts and behaviors, including 35% of autistic people (10% non‐autistic) who had previously attempted suicide. The mean total score of the 8 items on the PHQ‐9 was 12 for autistic people, which is above cutoff for possible clinical depression. The mean score on item 9 of the PHQ‐9 was 1 indicating that on average the autistic people in this sample had experienced suicidal thoughts for several days for the preceding 2 weeks. The mean score on each scale item was significantly higher for autistic than non‐autistic people.

**TABLE 2:** Individual items included in the analyses.

	Not autistic (*n* = 342) (mean (SD))	Autistic (*n* = 463) (mean (SD))	*p*	Results of goldbricker/nodename
GAD‐7				
1. Feeling nervous, anxious, or on edge	1.18 (0.96)	1.96 (1.04)	<0.01	Anxiety
2. Not being able to stop or control worrying	0.95 (0.98)	1.74 (1.09)	<0.01	Anxiety
3. Worrying too much about different things	1.01 (0.99)	1.82 (1.07)	<0.01	Anxiety
4. Trouble relaxing	1.17 (1.04)	1.98 (1.07)	<0.01	Relax
5. Being so restless that it is hard to sit still	0.57 (0.85)	1.36 (1.14)	<0.01	Movement
6. Being easily annoyed or irritable	1.00 (0.90)	1.66 (1.05)	<0.01	Annoy
7. Feeling afraid as if something awful might happen	0.67 (0.90)	1.49 (1.15)	<0.01	Anxiety
PHQ‐9				
1. Little interest or pleasure in doing things	0.79 (0.97)	1.45 (1.05)	<0.01	Interest
2. Feeling down, depressed, or hopeless	0.86 (0.98)	1.56 (1.10)	<0.01	Depressed
3. Trouble falling or staying asleep or sleeping too much.	1.30 (1.12)	1.89 (1.13)	<0.01	Sleep
4. Feeling tired or having little energy	1.34 (1.05)	1.97 (1.03)	<0.01	Tired
5. Poor appetite or overeating	0.99 (1.12)	1.69 (1.18)	<0.01	Appetite
6. Feeling bad about yourself—or that you are a failure or have let yourself or your family down	0.96 (1.09)	1.76 (1.14)	<0.01	Failure
7. Trouble concentrating on things, such as reading the newspaper or watching television	0.82 (0.98)	1.64 (1.18)	<0.01	Concentrate
8. Moving or speaking so slowly that other people could have noticed? Or the opposite—being so fidgety or restless that you have been moving around a lot more than usual?	0.25 (0.65)	0.87 (1.05)	<0.01	Movement
9. Thoughts that you would be better off dead, or hurting yourself in some way	0.33 (0.76)	0.97 (1.09)	<0.01	Dead
INQ‐10—Perceived burdensomeness				
1. The people in my life would be better off if I were gone.	1.73 (1.34)	2.87 (1.83)	<0.01	Better
2. The people in my life would be happier without me.	1.72 (1.33)	2.78 (1.78)	<0.01	Better
3. I think my death would be a relief to the people in my life.	1.48 (1.18)	2.45 (1.79)	<0.01	Rid
4. I think the people in my life wish they could be rid of me.	1.61 (1.28)	2.50 (1.65)	<0.01	Rid
5. I think I make things worse for the people in my life.	2.18 (1.67)	3.7 (2.03)	<0.01	Better
INQ‐10 Thwarted belonging				
6. I feel like I belong.	3.33 (1.95)	5.15 (1.69)	<0.01	Belong
7. I am fortunate to have many caring and supportive friends.	2.95 (1.92)	4.53 (1.94)	<0.01	Friends
8. I feel disconnected from other people.	3.39 (1.88)	5.14 (1.69)	<0.01	Belong
9. I feel like an outsider at social gatherings.	3.65 (1.89)	5.86 (1.47)	<0.01	Outsider
10. I am close to other people.	3.23 (1.86)	4.90 (1.69)	<0.01	Close

### Data splitting

As shown in Figure S1a, the data split primarily on autism diagnosis: the data differed significantly between those formally diagnosed, self‐diagnosed, and awaiting autism diagnosis (autistic) compared with those who had never considered they might be autistic (non‐autistic). The non‐autistic data then split significantly according to gender (male vs. non‐male), but there was no gender split in the autistic data. Thus, we proceeded with the analysis of autistic compared with non‐autistic people with combined gender groups.

### Item selection

As shown in Table 2, we combined items as follows: the belief that life would improve for others in the event of your death (“better”), combined items 1, 2, and 5, INQ‐10. Believing others aspire for your death (“rid”) combined items 3 and 4, INQ‐10. Not belonging (“belong”) combined items 6 and 8, INQ‐10. Movement differences (“movement”) combined item 8 PHQ‐9 (restlessness and shutdown) and item 5, GAD‐7 (restlessness); (v) anxiety, including uncontrollable worry, feeling nervous and believing something terrible will happen, (“anxiety”) combined items 1, 2, 3 and 7, GAD‐7. Items representing sleep (PHQ‐9 item 3) and appetite difficulties (PHQ‐9 item 5) were flagged but we retained these as distinct constructs.

1. **Is being autistic a distal risk marker for suicide? How does being autistic connect to other nodes to suggest pathways to suicidal thoughts?**

### Single network estimation

**FIGURE 2:**
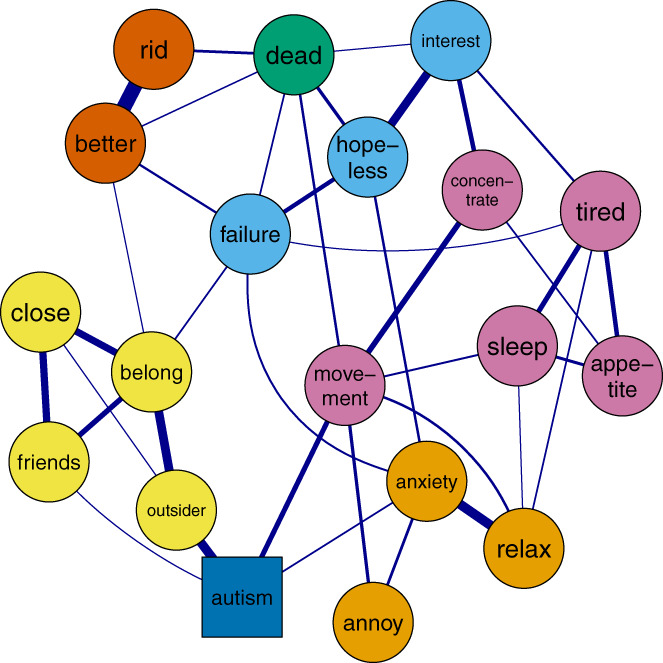
Whole sample network graph showing how being autistic connects with network nodes to thoughts of suicide and self‐harm. Note: Yellow = thwarted belongingness, light blue = low mood, green = thoughts of self‐harm or suicide, orange = anxiety, dark orange = perceived burdensomeness, pink = somatic experiences and dark blue = autism diagnosis. For full details and node names see Table 2. Graph minimum = 0.09, max = 0.14, cut = 0.09 for ease of interpretation. Theme = colorblind.

The network contained 138 edges between the 19 nodes. Eleven edges directly connected with thoughts of suicide and self‐harm. The strongest connections (shown in Figure 2) were with feeling depressed and hopeless (partial correlation, *r* = 0.19), believing others wish to be rid of you (*r* = 0.16), believing life would be better for others if you were gone (*r* = 0.16), feeling like a failure (*r* = 0.12) and movement differences (*r* = 0.14). Being autistic connected directly to feeling like an outsider, lacking caring and supportive friends, movement differences, and anxiety. Overall, this suggests that being autistic is a distal risk marker for suicide, which activates the network through anxiety, feeling like an outsider, lacking caring and supportive friends, and movement differences.

As shown in Figure 2, movement differences connected directly to thoughts of suicide and self‐harm. Feeling like an outsider and lacking caring and supportive friends connected to thoughts of suicide and self‐harm through feeling like you do not belong and feeling like a failure. Anxiety connected to thoughts of suicide and self‐harm through feeling depressed and hopeless. Overall, this suggests simultaneous pathways from autism diagnosis to thoughts of suicide and self‐harm through (i) movement differences; (ii) feeling like you do not belong, that others would be better off without you and feeling like a failure; (iii) anxiety and feeling depressed/ hopeless; and (iv) anxiety, somatic experiences, and low mood.

2 **Are the network structure and global strength consistent for autistic and non‐autistic people? How do connections between risk markers differ for autistic compared with non‐autistic people?**

### Joint network estimation

**FIGURE 3:**
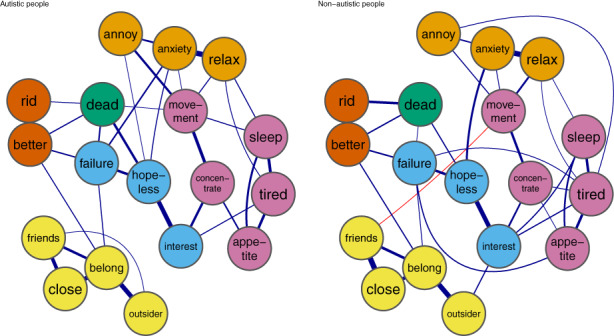
Network graphs for autistic (left) and non‐autistic (right) adults. Note: Yellow = thwarted belongingness, light blue = low mood, green = thoughts of self‐harm or suicide, orange = anxiety, dark orange  = perceived burdensomeness, pink = somatic experiences. For full details and node names, see Table 2. Graph minimum = 0.08, max = 0.95, cut = 0.08 for ease of interpretation.Theme = colorblind. We use the *averagelayout* function to provide a consistent layout for networks.

There were consistent edges in the autistic (*n* = 160) and non‐autistic (*n* = 162) between 18 nodes with strongest connections shown in Figure 3. In both networks, nine nodes connected to thoughts of suicide and self‐harm and in both groups the strongest edges were with feeling hopeless (autistic *r* = 0.21, non‐autistic *r* = 0.13) and believing others would be better off if you were gone (autistic *r* = 0.12, non‐autistic *r* = 0.14). In the autistic group, strongest edges also included feeling like a failure (*r* = 0.15), and in the non‐autistic group, believing others wish to be rid of you (*r* = 0.22).

### Network comparisons

The Network Comparison Test (NCT) reported that global density was greater in the non‐autistic network (global density = 8.47) than in the autistic network (global density = 8.30), but this was not statistically significant (*p* = 0.30).

Eight edge strengths differed significantly between autistic and non‐autistic networks. First, four edges connected mood symptoms with somatic experiences for the non‐autistic network but were absent in the autistic network. These edges connected: (i) losing interest and sleep difficulties (autistic network *r* = 0, non‐autistic network *r* = 0.36, *p* = 0.02), (ii) feeling annoyed and feeling tired (autistic network *r* = 0, non‐autistic network *r* = 0.10, *p* < 0.01), (iii) feeling like a failure and appetite difficulties (autistic network *r* = 0, non‐autistic network *r* = 0.23, *p* = 0.02), and (iv) sleep difficulties and thoughts of self‐harm and ending life (autistic network *r* = 0, non‐autistic network *r* = 0.04, *p* = 0.01). Overall, this suggests mood symptoms are associated with somatic experiences for non‐autistic people but are independent for autistic people.

Second, three edges connected thwarted belonging experiences with mood symptoms or burdensomeness for non‐autistic people but were absent for autistic people. These were edges between: (i) feeling like an outsider with (a) feeling annoyed (autistic network = 0, non‐autistic network = 0.07, *p* = 0.05) and (b) losing interest (autistic network = 0, non‐autistic network = 0.04, *p* = 0.03); (ii) lacking caring and supportive friends and believing other would be better off if you were gone (autistic network = 0, non‐autistic network = 0.04, *p* = 0.02). Furthermore, (iii) not feeling close to other people was negatively connected to having difficulty relaxing (autistic network = −0.02, non‐autistic = 0, *p* = 0.04) in autistic people but unconnected in non‐autistic people. Overall, this suggests that thwarted belonging is independent of, or differently connected to affective symptoms or burdensomeness for autistic compared with non‐autistic people.

**FIGURE 4:**
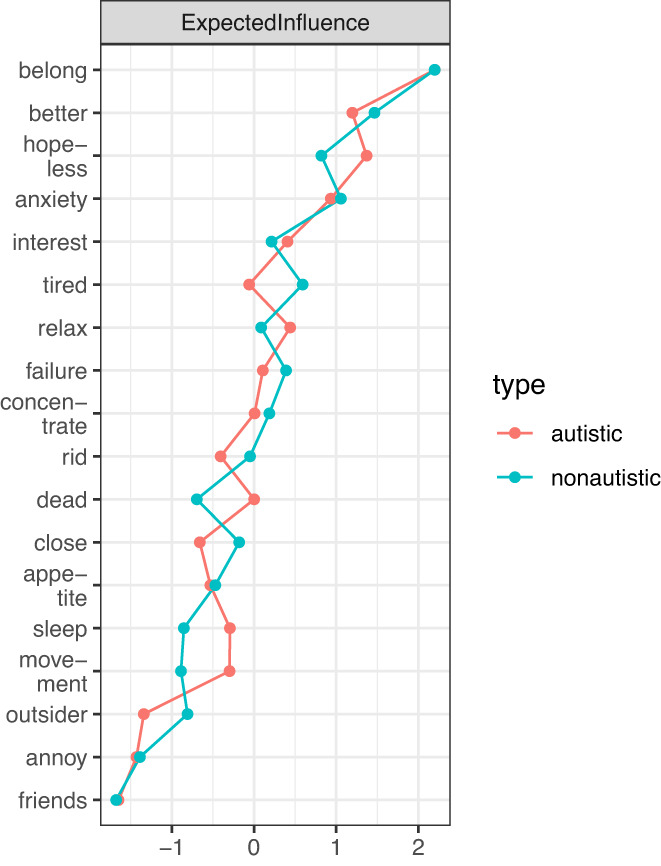
Standardized relative expected influence of nodes within autistic and non‐autistic networks.

As shown in Figure 4, similar nodes were most influential in each group; the 75th percentile expected influence nodes in both groups were as follows: (1) feeling like you do not belong, (2) feeling depressed, (3) believing others wish you were gone, (4) feeling anxious, and (5) having difficulty relaxing (autistic network) and feeling tired (non‐autistic network). Unstandardized expected influence is reported in Figure S1b. Expected Influence was similar in each network, however, two nodes were significantly more inter‐connected in the non‐autistic than autistic networks. Feeling like an outsider (total sum of edges on this node in autistic network = 0.60, non‐autistic network = 0.78, *p* = 0.01) and feeling tired (autistic network = 0.88, non‐autistic network = 1.03, *p* = 0.05) had significantly greater expected influence in the non‐autistic than autistic networks.

### Network stability

As shown in Figures S2–S7, the edge weights were accurately measured. The centrality stability coefficient was 0.52 in the non‐autistic network and 0.75 in the autistic network suggesting that expected influence scores were stable.

## DISCUSSION

For the first time, we used network analysis to extend our theoretical understanding of how anxiety and depression contribute to suicidal thoughts for autistic and non‐autistic adults. In line with our expectations, autistic adults reported more frequent suicidal thoughts, anxiety, depression, thwarted belonging, and perceived burdensomeness than non‐autistic adults. Exploratory analyses reported that being autistic connected directly to feeling like an outsider, lacking caring and supportive friends, anxiety, and movement (such as restlessness). These experiences connected to suicidal thoughts through feelings of low mood (hopelessness or failure) and burdensomeness (believing others wish you were gone), except movement, which connected directly to suicidal thoughts. Group difference tests reported that overall inter‐connectedness was consistent for autistic and non‐autistic networks. In the non‐autistic network, mood symptoms connected to thwarted belonging and somatic experiences, but these were independent in the autistic network. Feeling tired and like an outsider were less inter‐connected for autistic than for non‐autistic adults.

In line with previous research, autistic adults reported more frequent suicidal thoughts (Cassidy et al., 2014), burdensomeness (Camm‐Crosbie et al., 2019; Pelton et al., 2020b), unmet need for belonging (Milton & Sims, 2016), anxiety and depression (Lever & Geurts, 2016) than non‐autistic adults. In line with the network approach, there were multiple interactions within and between items designed to measure distinct constructs (Borsboom, 2017; Borsboom & Cramer, 2013; Cramer et al., 2010) and more experiences connected to suicidal thoughts than epidemiology studies suggest (De Beurs, 2017; De Beurs et al., 2017, 2021). Overall, this suggests network analysis is a helpful transdiagnostic alternative to research boundaried by non‐autistic categories (Lombardo et al., 2019; Weiss, 2014) to extend our understanding of the development of suicidal thoughts and behaviors.

**FIGURE 5:**
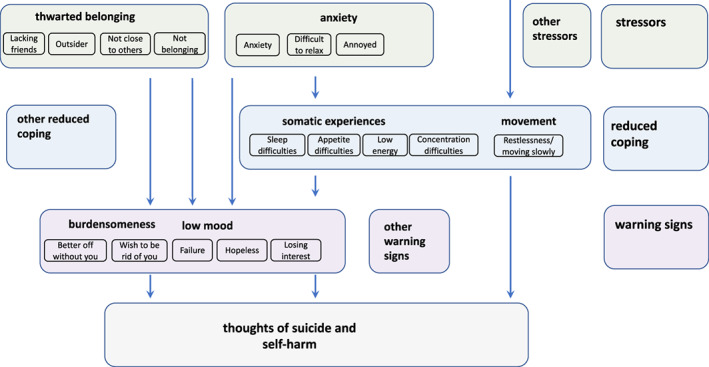
Conceptual model of progression from environmental stressors, reduced coping, and warning signs to suicidal thoughts.

Our results describe, for the first time, how being autistic may be a distal risk marker for suicide. As described in conceptual model Figure 5, being autistic is connected directly to daily stressors, feeling like an outsider, lacking friends, and anxiety in line with research describing that autistic adults report loneliness (Causton‐Theoharis et al., 2009; Hedley, Uljarević, Wilmot, et al., 2018), feeling “othered” (being treated as intrinsically different or inhuman) (Cage et al., 2019; Michael, 2021) and experience significant daily anxiety (Uljarević et al., 2020), which negatively impacts quality of life (Mason et al., 2018). In line with previous research, there was no connection between anxiety and thwarted belonging (Rath et al., 2019). Anxiety is connected with somatic experiences, such as sleep difficulties, which is a particular concern for autistic people in the development of suicidal thoughts and behaviors (Cassidy, Cogger‐Ward, et al., 2021). In our network, sleep was strongly inter‐connected with tiredness and appetite, suggesting inter‐dependency, and linked with movement and concentration difficulties. This could suggest anxiety erodes somatic coping mechanisms to decrease mood. Thwarted belonging connected to feelings of burdensomeness as in previous network analyses (De Beurs et al., 2019) and to feeling like a failure: a more nuanced description of how thwarted belonging contributes to depression (Kleiman et al., 2014), resonating with “failed social struggle” described by the Integrated Motivational Volitional model of suicide (O'Connor, 2011). Both burdensomeness items (believing others are better off without you/wish to be rid of you), feeling like a failure, hopeless, and losing interest in life connected closely and directly to suicidal thoughts, suggesting these are potent warning signs of those most at risk. Thus, in sum, as shown in Figure 5, our results suggest a progression from daily stressful or traumatic experiences through reduced coping and warning signs to suicidal thoughts.

The connection between being autistic and movement reflects the long‐standing role of repetitive behaviors as a diagnostic criterion. More surprising is the direct connection from movement to suicidal thoughts though one study reported agitation as a unique risk marker for suicide attempts in individuals with high autistic traits (Takara & Kondo, 2014). One possibility is that other experiences, such as sensory overload, may be coped with through movement and/or other warning signs that mediate the relationship with suicidal thoughts. However, “stimming” (self‐stimulation) is important for autistic people to regulate uncontainable emotions (Kapp et al., 2019; Pearson & Rose, 2021) so future partnership research could explore the meaning of our findings and how stimming and movement regulate emotional distress.

Our model allows interventions to be identified at each level; for example, understanding and reducing anxiety (Parr et al., 2020), promoting belonging (Milton & Sims, 2016) and autism acceptance (Cage et al., 2018) could reduce the extent to which the network is activated. Maintaining relationships (Hedley, Uljarević, Foley, et al., 2018; Hedley, Uljarević, Wilmot, et al., 2018), peer relationships (Cage et al., 2022), enabling a healthy lifestyle, and meeting support needs (Cassidy, Bradley, Shaw, et al., 2018) could prevent the progression from stressors to warning signs. Identifying hopelessness, burdensomeness, loss of interest in life and failure could be incorporated into safety planning/risk assessments to improve crisis care (Schwartzman et al., 2021). Our model extends the ITS suggesting that, in addition to burdensomeness, low mood is proximal and significant in the development of suicidal thoughts. The distal role of thwarted belonging reflects previous network analyses (De Beurs et al., 2019; Ordóñez‐Carrasco et al., 2021) and the protective role of connectedness outlined in the 3Step theory (Klonsky & May, 2015). Our results emphasize the role of burdensomeness as a proximal risk marker, but failure and hopelessness also resonate with broader emotional pain proposed by the Three‐step Theory (3ST) (Klonsky & May, 2015). Caution should be exercised that our model includes only a subset of risk markers and those at greatest risk of suicide are likely to experience a wide range of risk markers (Cassidy et al., 2022).

However, before pursuing future research, we should consider the comparison between autistic and non‐autistic networks. Overall connectedness was consistent between autistic and non‐autistic networks, but connections from mood symptoms with somatic and thwarted belonging experiences were absent for autistic adults, and feeling tired and like an outsider were significantly less inter‐connected in the autistic than non‐autistic network. These results suggest that somatic experiences described in the PHQ‐9 and GAD‐7 are not the most pertinent to capture emotional change for autistic people. New tools to measure depression and anxiety have identified more precise somatic indicators for autistic adults, such as a *change* in fatigue, rather than the *presence* of fatigue (Cassidy, Bradley, Cogger‐Ward, et al., 2021) and anxiety indicators, such as *feeling shaky* (Rodgers et al., 2016, 2020). Similarly, *feeling like an outsider* has already been identified as a less meaningful indicator of thwarted belonging for autistic than for non‐autistic adults (Pelton et al., 2020a). Thus, our model takes an important first step in demonstrating how autistic adults experience general population risk markers but, may not yet capture unique risk markers for autistic adults. Additional stressors could include uncertainty (Boulter et al., 2014), pressures contributing to autistic burnout (Higgins et al., 2021; Mantzalas et al., 2022; Raymaker et al., 2020), and research could explore how the experience of those stressors is influenced by age (Stewart et al., 2022) or gender (Kõlves et al., 2021) for autistic people to contribute to unique risk.

Our study highlights methodological challenges in comparing suicidal thoughts and behaviors for autistic and non‐autistic adults. First, drawing on non‐clinical samples, autistic adults report shockingly more frequent experiences of depression, suicidal thoughts, and behaviors than non‐autistic adults; researchers should carefully consider recruitment and statistical methods for accurate comparison. Second, our findings suggest we may be reaching the limits of what we can infer about suicidal thoughts and behaviors for autistic adults using constructs and measurement tools designed for non‐autistic adults. Commentary argues that research should focus on *relevant* experiences for autistic people rather than *statistically valid* non‐autistic measurement tools (Jones, 2022) reflecting more general concerns about precise construct definition and accurate measurement for suicide and psychological theory (Bringmann et al., 2022; Lawson & Robins, 2021; Millner et al., 2020). Overall, this could suggest that future theory development take autistic lived experience as its starting point and articulate a model independent of non‐autistic experience.

This study has several strengths: this is the first study to apply network analysis to explore suicide theory in autistic people responding to calls to go beyond single risk factor studies and apply novel, rigorous methods in a transdiagnostic approach (Franklin et al., 2017; Lombardo et al., 2019; Millner et al., 2020). The development of support, interventions, risk assessments, and crisis services for autistic adults (Cassidy, Cogger‐Ward, et al., 2021; Jager‐Hyman et al., 2020) is essential to meet global suicide prevention goals. This study is limited by an absence of valid measurement tools to explore suicidal thoughts and behaviors for autistic people at the time of design. Researchers should consider using the recently published Suicidal Behaviours Questionnaire‐Revised (Autism Spectrum Condition) (SBQ‐ASC) (Cassidy, Bradley, Cogger‐Ward, & Rodgers, 2021) or the Suicidal Ideation Attributes Scale‐Modified (SIDAS‐M) (Hedley et al., 2022). This study used self‐report autism diagnosis, which falls short of “gold standard” confirmation of autism but allows for wider participation. The sample size is relatively small (see van Borkulo et al., 2022 for discussion on appropriate sample size) for group comparison network analyses); thus, future research could employ cohort, population studies, or simulation studies to replicate and extend our findings. Furthermore, our cross‐sectional design cannot infer causality; thus, findings should be considered strictly exploratory, and taking a time‐series or longitudinal approach should confirm intervention targets (Rath et al., 2019). Future network analyses could, thus, apply temporal analyses (Haslbeck & Waldorp, 2015) drawing on ecological momentary assessment (Rath et al., 2019), moderation analysis to explore how autistic characteristics (such as difficulty switching attention) moderate connections between risk markers (Haslbeck, Borsboom, et al., 2021) or cluster analysis to accurately define and measure constructs (Forkmann et al., 2018; Golino & Epskamp, 2017).

## CONCLUSION

This study reports that being autistic represents a unique distal risk marker for suicidal thoughts and behaviors through feeling like an outsider, anxiety, and movement differences. Research should extend these exploratory findings using longitudinal study designs in partnership with autistic people, ensuring constructs are meaningfully defined to produce suicide theory that accurately reflects lived experience of autistic people. This will be vital to ensure tailored suicide prevention for autistic adults.

## Supporting information


Appendix S1

Figure S1

Figures S2–S7

## Endnote

^1^
We refer to ‘autistic people’ (termed ‘identity‐first language’) to reflect the published preference of the majority of autistic people that autism is an integral component of identity (Kenny et al., 2016). We avoid the use of ‘person‐first language’ (such as ‘person with autism’), which has been associated with stigma and dehumanization (Botha et al., 2021). For guidance on language choices in autism research see (Bottema‐Beutel et al., 2021).
