# Supplementary material for: The role of anxiety and depression in suicidal thoughts for autistic and non‐autistic people: A theory‐driven network analysis
Source: Suicide Life Threat Behav. 2023 Mar 28;53(3):426–42. doi: 10.1111/sltb.12954 (PMC10947106; doi:10.1111/sltb.12954)
Supplement: Supplementary file 1 — Appendix S1 [file SLTB-53-426-s001.docx]

**Supplementary 1a: Technical details of the analysis**

Statistical analyses were conducted in R (version 4.0.5; R core team 2021).

**Exploring the data:** Item distributions were kurtotic as they intend to capture rare experiences (van Orden et al 2008) so data were transformed using the r package *huge* to relax the expectation of normality. Items were on an ordinal scale so we used Spearman correlations using the cor_auto function in qgraph. We used the *networktree* package to explore the most influential split within the data based on autism diagnosis (autistic, not autistic, seeking diagnois, not seeking diagnosis) and gender (female, male, not male or female).

**Item selection**: Over-lapping constructs pose a problem for network inference so steps should be taken to ensure that nodes represent distinct conceptual entities (Burger et al 2021; Fried & Cramer 2017). There is currently no *best practice* solution available, thus, we drew on the work of others (e.g. Lass et al 2020; Barthel et al 2020) and used the *goldbricker* function from the *networktools* R package (Jones 2017) to compare correlations between network nodes and flag pairs where few correlations differ. We considered whether flagged pairs: (i) were distinct constructs and should be retained; (ii) over-lapped entirely and thus, one item should be removed; or (iii) over-lapped partially and, thus, items should be combined using the *reduce_net* function from the *networktools* package which combines items using a principal component analysis. We used theoretical understanding, network visualisation and adjacency matrices to inform decisions.

**Whole sample network estimation:** We used the *Mixed Graphical Models (mgm)* package to estimate the network in the whole sample with the continuous variables measuring individual items and the categorical variable autism diagnosis. We set a random seed at the outset to ensure the analysis is fully reproducible and applied a cross-folds validation with 10 folds as penalization. The partial correlations between each node and others in the network are equivalent to regression coefficients in a regression model, thus, nodes can mediate one another in a putative causal, mediating relationship (as in Isvoranu et al 2017). For a tutorial on *mgm* see (Haslbeck, Jonas & Waldorp, 2015).

**Group network estimation:** We estimated networks in autistic and non-autistic people using the *Estimate Group Network* package, which allows for greater statistical power by combining datasets and improves network estimates by exploiting similarities across groups (Danaher et al 2014). If this does not improve model fit, separate networks are estimated, which more accurately identify similarities and differences across groups than individually estimated networks. We report here the model employing Extended Bayesian Information Criterion (EBIC) as regularization. For a tutorial, see (Constantini et al 2017).

**Network visualization:** We visualised networks using the r *qgraph* package (Epskamp, Cramer, Waldrop, Schmittmann & Borsboom 2012). The Fruchterman-Reingold (FR) algorithm is the most frequently used placing algorithm (Jones, Mair & McNally 2018) and places highly connected nodes towards the centre and less connected nodes to the periphery with as few crossing edges as possible (Fruchterman and Reingold 1991). Edge width is directly proportional to the absolute value of the edge weight with wider edges representing stronger edges.

**Expected influence:** We selected *expected influence* as centrality estimate (total sum of edge weights on a given node taking into account negative associations between nodes) (Robinaugh, Millner & McNallly 2016) as other centrality measures may be less reliably estimated (Epskamp et al 2017).

**Network comparison:** We calculated the correlation co-efficient between the two networks and the largest absolute differences in edge weights and expected influence between the autistic and non-autistic networks. Next, we used the *Network Comparison Test* (van Borkulo, Epskamp & Milner 2016) to test whether networks are statistically invariant with respect to global strength, individual edge weights and expected influence. For tutorial see (van Borkulo 2018).

**Network stability:** we used the *estimatebootnet* function in *bootnet* to estimate the accuracy of network edge estimates and stability of centrality indices. For tutorial see (Epskamp et al., 2018).

Supplementary 1b: r script for the analysis

## install packages

library("mgm")

library("qgraph")

library("bootnet")

library("networktools")

library("haven")

library("networktree")

library("EstimateGroupNetwork")

library("stringr")

library("networktree")

library("igraph")

library("NetworkComparisonTest")

library("psychTools")

library("summarytools")

library("huge")

library("psych")

library("cyphr")

library("rockchalk")

library("dplyr")

## this is the script for the published paper.

## The shared data includes only a subset of the

## published data as some participants withheld consent for data sharing.

## This script will need to be adjusted accordingly.

inqplusdata<-read.csv('2804_inqplusdata.csv', header=TRUE)

## set categorical variables

inqplusdata$AUTISM_DIAG<-as.factor(inqplusdata$AUTISM_DIAG)

inqplusdata$AUTISM_DIAG <- factor(inqplusdata$AUTISM_DIAG,

levels = c(1,2,3,4),

labels = c("Not autistic",

"Not seeking diagnosis",

"Awaiting diagnosis",

"Autistic"))

inqplusdata$GENDER<-as.factor(inqplusdata$GENDER)

inqplusdata$GENDER <- factor(inqplusdata$GENDER,

levels = c(1,2,3,4),

labels = c("Male",

"Female",

"Not male or female",

"Prefer not to say"))

## check totals each category

table(inqplusdata$AUTISM_DIAG)

table(inqplusdata$GENDER)

############################################################

######### prepare data: networktree script #################

############################################################

## to explore where the most influential splits in the data are

## we use the network tree package which uses recursive partitioning

## to identify influential splits in the data on an exploratory basis

## we do this because of systematic bias in access to autism diagnosis

## thus, we ask whether those who think they might be autistic

## are statistically similar to those with or without diagnosis

## select variables for tree analysis

treeData<-subset(inqplusdata, select=c("depression",

"anxiety",

"belonging","burden",

"AUTISM_DIAG",

"GENDER", "AGE_1",

"Q455", "T1_PHQ9_DEAD"))

## select variables for initial tree analysis

## split variables are gender and autism diagnosis status

tree1<-subset(treeData, select=c("depression",

"anxiety","belonging",

"burden", "T1_PHQ9_DEAD",

"AUTISM_DIAG",

"GENDER"))

## check counts in each group

mytable_autism<-table(tree1$AUTISM_DIAG)

mytable_autism

mytable_gender<-table(tree1$GENDER)

mytable_gender

## create correlation matrix

cor_tree<-cor_auto(tree1[,c(1:5)])

## create labels

tree_names<-c("Dep", "Anx",

"Bel", "Bur", "Sui")

## visualise network

q1<-qgraph(cor_tree, layout="spring", labels=tree_names)

## specify node variable and split variables

tree_nodevars<-tree1[,1:5]

tree_splitvars<-tree1[,6:7]

## run network tree

cor_aut2<-networktree(nodevars=tree_nodevars, cor="cor_auto",

splitvars=tree_splitvars, method="ctree",

transform="glasso")

# print function allows us to view network

print(cor_aut2)

## create average layout of all non-autistic male and female and

## autistic combined

no_aut_female<-getnetwork(cor_aut2, id=4)

no_aut_male<-getnetwork(cor_aut2, id=3)

aut_network<-getnetwork(cor_aut2, id=5)

no_aut_network<-getnetwork(cor_aut2, id=2)

R<-qgraph(no_aut_female, layout="spring")

S<-qgraph(no_aut_male, layout="spring")

U<-qgraph(aut_network, layout="spring")

V<-qgraph(no_aut_network, layout="spring")

laYout<-averageLayout(U,V)

## visualize network

pdf("1601_networktree.pdf", height = 15, width=25)

plot(cor_aut2, edge.labels=TRUE, minimum=0.1, cut=0.06,

edge.label.cex=1.5, labels=tree_names,label.fill.vertical=1,

partyargs = list(gp=grid::gpar(cex=2)),

theme="colorblind", layout=laYout)

dev.off()

########################################################################

## select variables and data screening ##############################################

########################################################################

## select variables

anxdep_cluster<-subset(inqplusdata,

select = c("T1_GAD1_NERVOUS",

"T1_GAD2_WORRYSTOP",

"T1_GAD3_WORRY", "T1_GAD4_RELAX",

"T1_GAD5_RESTLESS", "T1_GAD6_ANNOY",

"T1_GAD7_AWFUL",

"T1_PHQ1_INTEREST",

"T1_PHQ2_HOPeLESS", "T1_PHQ3_SLEEP",

"T1_PHQ4_ENERGY", "T1_PHQ5_APPETITE",

"T1_PHQ6_FAILURE",

"T1_PHQ7_CONCENTRATE",

"T1_PHQ8_MOVESLOW", "T1_PHQ9_DEAD",

"T1_INQ_1_GONE", "T1_INQ_2_HAPPIER",

"T1_INQ_3_RELIEF", "T1_INQ_4_RID",

"T1_INQ_5_WORSE", "T1_INQ_6_BELONG",

"T1_INQ_7_CARING", "T1_INQ_8_DISCON",

"T1_INQ_9_OUTSIDER", "T1_INQ_10_CLOSE",

"AUTISM_DIAG"))

########################################################################

## prepare data ##############################################

########################################################################

## check alphas

GAD7<-data.frame(anxdep_cluster[,1:7])

PHQ9<-data.frame(anxdep_cluster[,8:16])

INQ_TB<-data.frame(anxdep_cluster[,17:21])

INQ_PB<-data.frame(anxdep_cluster[,22:26])

alpha(GAD7)

alpha(PHQ9)

alpha(INQ_TB)

alpha(INQ_PB)

## remove missing cases, transform non-normal data

## explores for overlapping constructs and combines possibly autistic

## and autistic people

## retain complete cases

data_subset<-anxdep_cluster[,1:27]

anxdep_cluster1<-anxdep_cluster[complete.cases(data_subset), ]

## check missing data

is.na(anxdep_cluster1)

sum(is.na(anxdep_cluster1))

## explore and transform data

## transform data apart from autism diagnosis category

anxdep_cluster1[,-27]<-huge.npn(anxdep_cluster1[,-27])

## check again the type of data

str(anxdep_cluster1)

# rename to be sure this is transformed data

anxdeptrans<-anxdep_cluster1

###############################################################################

########## check for overlapping constructs using goldbricker ###############

###############################################################################

gb_all<-goldbricker(anxdeptrans[,-27], p = 0.05,

method = "hittner2003", threshold = 0.25,

corMin = 0.5, progressbar = TRUE)

gb_all

badPairs1<-c("T1_GAD3_WORRY", "T1_GAD2_WORRYSTOP",

"T1_INQ_4_RID", "T1_INQ_3_RELIEF",

"T1_INQ_8_DISCON", "T1_INQ_6_BELONG",

"T1_PHQ8_MOVESLOW", "T1_GAD5_RESTLESS",

"T1_INQ_2_HAPPIER", "T1_INQ_1_GONE",

"T1_GAD7_AWFUL", "T1_GAD1_NERVOUS")

## run reducenet function to combine items using PCA

anxdeptrans1<-net_reduce(anxdeptrans,

badpairs = badPairs1,

method=c("PCA"))

## run a second round of goldbricker

gb_all1<-goldbricker(anxdeptrans1[,-15], p = 0.05,

method = "hittner2003", threshold = 0.25,

corMin = 0.5, progressbar = TRUE)

gb_all1

## this brings up further 'bad pairs' in burdensomeness and anxiety items

badPairs2<-c("PCA.T1_INQ_2_HAPPIER.T1_INQ_1_GONE", "T1_INQ_5_WORSE",

"PCA.T1_GAD7_AWFUL.T1_GAD1_NERVOUS",

"PCA.T1_GAD3_WORRY.T1_GAD2_WORRYSTOP")

## run reducenet to combine again because these overlap

anxdeptrans2<-net_reduce(anxdeptrans1,

badpairs = badPairs2,

method=c("PCA"))

## run 3rd goldbricker on version 3

gb_all2<-goldbricker(anxdeptrans2[,-14], p = 0.05,

method = "hittner2003", threshold = 0.25,

corMin = 0.5, progressbar = TRUE)

gb_all2

## this time just returns PHQ5 and 3 appetite and sleep which qre distinct so

## proceed with next step

## next combine levels as in network tree analysis

anxdeptrans2$AUTISM_DIAG<-combineLevels(anxdeptrans2$AUTISM_DIAG,

c(2,3,4),

newLabel =

"Autistic")

## then rename variables to make it all a bit more manageable using dplyr

anxdeptrans2<-anxdeptrans2 %>%

rename(relax=T1_GAD4_RELAX,

annoy=T1_GAD6_ANNOY,

interest=T1_PHQ1_INTEREST,

depressed=T1_PHQ2_HOPeLESS,

sleep=T1_PHQ3_SLEEP,

energy=T1_PHQ4_ENERGY,

appetite=T1_PHQ5_APPETITE,

failure=T1_PHQ6_FAILURE,

concentrate=T1_PHQ7_CONCENTRATE,

dead=T1_PHQ9_DEAD,

friends=T1_INQ_7_CARING,

outsider=T1_INQ_9_OUTSIDER,

close=T1_INQ_10_CLOSE,

autism=AUTISM_DIAG,

rid=PCA.T1_INQ_4_RID.T1_INQ_3_RELIEF,

belong=PCA.T1_INQ_8_DISCON.T1_INQ_6_BELONG,

motor=PCA.T1_PHQ8_MOVESLOW.T1_GAD5_RESTLESS,

gone=PCA.PCA.T1_INQ_2_HAPPIER.T1_INQ_1_GONE.T1_INQ_5_WORSE,

anxiety=PCA.PCA.T1_GAD7_AWFUL.T1_GAD1_NERVOUS.PCA.T1_GAD3_WORRY.T1_GAD2_WORRYSTOP)

View(anxdeptrans2)

## relocate autism diagnosis to end to make it easier to run mgm

anxdeptrans2<-anxdeptrans2 %>%

relocate(autism, .after=anxiety)

#######################################################################

##################### whole sample mgm network ##################

#######################################################################

## recode autism as numeric for mgm network

anxdeptrans2$autism<-as.numeric(anxdeptrans2$autism)

## run mgm network to examine influence of

## categorical autism diagnosis on whole sample

## set type and level

str(anxdeptrans2)

tyPe<-c(rep("g", 18),"c")

leVel<-c(rep(1,18),2)

set.seed(168)

mgmwhole<-mgm(data=anxdeptrans2, type=tyPe, level=leVel,

lambdaFolds=10,

lambdaSel = "CV",

k=2,

scale=TRUE)

## it is a 19x19 matrix so total of 361

## how many edges are zero

mgmwhole$pairwise$wadj

colSums(mgmwhole$pairwise$wadj !=0) ## 138 edges are generated

## visualise network

laBels_whole<-c("relax", "annoy", "interest", "hope-\nless", "sleep",

"tired", "appe-\ntite", "failure", "concen-\ntrate",

"dead", "friends", "outsider", "close",

"rid", "belong", "move-\nment", "better", "anxiety", "autism")

## name groups

groUps.whole<-list(c(1:2,16,18), c(3:9, 16), c(10), c(11:13, 15),

c(14,17), c(19))

groUps.whole1<-list(c(1:2,18), c(3:4, 8), c(10), c(11:13, 15), c(5:7, 9, 16),

c(14,17), c(19))

names(groUps.whole1) <-

c("Anxiety", "Low mood", "Suicidal thoughts",

"Thwarted belonging", "Somatic", "Perceived burdensomeness",

"Autism diagnosis")

## curve instructions need to be adjusted because they relate to the full

## dataset and we have only consent to share certain records

curve.whole<-c(14, 38, 65, 59)

curve.rel<-(rep(0, 69))

curve.rel[curve.whole]<-c(0.5, -0.9, -0.4, -1.5)

## this is setting autism diagnosis as a square

shape.whole=c("circle", "circle","circle","circle",

"circle","circle","circle","circle",

"circle","circle","circle","circle",

"circle","circle","circle","circle",

"circle","circle", "square")

## visualise network

pdf("1601_wholenetwork1.pdf")

wholesample <- qgraph(mgmwhole$pairwise$wadj,

theme="colorblind", layout="spring",

repulsion=0.9, groups=groUps.whole1,

legend=FALSE, labels=laBels_whole,

vsize=10, shape=shape.whole,

minimum=0.09, cut=0.09,

maximum=0.14, curve = curve.rel)

dev.off()

## rerun without curvature to measure centrality

pdf("1601_wholenetwork_nocurve.pdf")

wholesample <- qgraph(mgmwhole$pairwise$wadj,

title="Whole sample",

theme="colorblind", layout="spring",

repulsion=0.9,

legend=FALSE, labels=laBels_whole,

vsize=10, shape=shape.whole,

minimum=0.09, cut=0.09,

maximum=0.14)

dev.off()

## this shows shortest pathway but it's a bit redundant because the shortest

## pathway is so obvious

pdf("1601_wholesample_pathways.pdf")

patHways<-pathways(wholesample, from = "autism",

to = "dead")

dev.off()

## test centrality of wholesample plot

## again, this is a bit redundant

pdf("1601_centrality whole.pdf")

centralityPlot(wholesample, include=c("Strength"), orderBy = "Strength")

dev.off()

## have a look at the edge list

wholesample$Edgelist

## check stability of this network through resampling

laBels_whole_res<-c("relax", "annoy", "interest", "depressed", "sleep",

"tired", "appetite", "failure", "concentrate",

"dead", "friends", "outsider", "close",

"rid", "belong", "motor", "better", "anxiety", "autism")

set.seed(1)

res_mgmwhole<-resample(object=mgmwhole, data=anxdeptrans2, nB=50)

## visualise bootstraps

pdf("1601_whole sample resample.pdf", height=20, width = 10)

plotRes(res_mgmwhole, axis.ticks = c(-.2, -.1, 0, .1, .2, .3, .4, .5, .6, .7, .8),

cex.label=0.5, labels=laBels_whole_res,

layout.width.labels = .40, cex.mean=0.3, cex.bg=1.6)

dev.off()

###############################################################################

################## group difference network using estimategroupnet ####

###############################################################################

## create separate datasets

anxdep_no_aut<-subset(anxdeptrans2,

autism == 1)

anxdep_aut<-subset(anxdeptrans2,

autism == 2)

## remove autism diagnosis variable

anxdep_aut<-anxdep_aut[-19]

anxdep_no_aut<-anxdep_no_aut[-19]

## run simple networks in each group using bootnet

aut_net <- estimateNetwork(anxdep_aut,

default="EBICglasso",

corMethod="cor_auto")

no_aut_net <- estimateNetwork(anxdep_no_aut,

default="EBICglasso",

corMethod="cor_auto")

## plot individual networks

plot(no_aut_net)

plot(aut_net)

## create average layout based on these graphs

laYout<-averageLayout(aut_net, no_aut_net)

## create labels for network graphs

laBels_group<-c("relax", "annoy", "interest", "hope-\nless", "sleep",

"tired", "appe-\ntite", "failure", "concen-\ntrate",

"dead", "friends", "outsider", "close",

"rid", "belong", "move-\nment", "better", "anxiety")

## create groups

groUps<-list(c(1:2,18), c(3:4, 8), c(10), c(11:13, 15), c(5:7, 9, 16),

c(14,17))

names(groUps) <-

c("Anxiety", "Low mood", "Suicidal thoughts",

"Thwarted belonging", "Somatic", "Perceived burdensomeness")

## plot individual networks

pdf("1601_single_group_network.pdf", width=10, height=5)

par(mfrow=c(1,2))

aut_plot <- plot(aut_net, layout=laYout,

title="Possibly/ autistic group single",

theme="colorblind", labels = laBels_group,

groups=groUps,

border.width=2, vsize=9, maximum=0.46,

border.color='#555555', legend=FALSE) # max= 0.46 - set graph max

noaut_plot <- plot(no_aut_net, layout=laYout,

title="Non-autistic group single", labels = laBels_group,

theme="colorblind",

border.width=2, vsize=9, groups=groUps,

border.color='#555555', legend=FALSE, maximum=0.46) ## maximum is 0.44

dev.off()

## check stability of individually estimated networks and centrality estimates

## Estimate and save stability and accuracy

boot_aut1a <- bootnet(aut_net, nBoots = 1000, nCores = 8)

boot_noaut2a <- bootnet(no_aut_net, nBoots = 1000, nCores = 8)

### Plot edge weight CI

pdf("1601_Edge weight CIs_aut.pdf")

plot(boot_aut1a, labels = FALSE, order = "sample")

dev.off()

pdf("1601_Edge weight CIs_noaut.pdf")

plot(boot_noaut2a, labels = FALSE, order = "sample")

dev.off()

### Edge weights diff test

pdf("1601_edge_weight diff_aut.pdf")

plot(boot_aut1a, "edge", plot = "difference",

onlyNonZero = TRUE, order = "sample", labels=FALSE)

dev.off()

pdf("1601_edge_weight diff_noaut.pdf")

plot(boot_noaut2a, "edge", plot = "difference",

onlyNonZero = TRUE, order = "sample", labels=FALSE)

dev.off()

### Centrality diff test

pdf("1601_centrality difference_aut.pdf")

plot(boot_aut1a, "strength", order="sample", labels=FALSE)

dev.off()

pdf("1601_centrality difference_noaut.pdf")

plot(boot_noaut2a, "strength", order="sample", labels=FALSE)

dev.off()

## centrality stability

Centrality_stability_aut<-bootnet(aut_net, nBoots = 1000,

type = "case",

nCores=8,

statistics = c("strength",

"betweenness",

"closeness",

"expectedInfluence"))

Centrality_stability_no_aut<-bootnet(no_aut_net, nBoots = 1000,

type = "case",

nCores=8, statistics = c("strength",

"betweenness",

"closeness",

"expectedInfluence"))

## plot centrality stability

pdf("1601_centrality_stability_aut.pdf")

plot(Centrality_stability_aut,

statistics = c("strength", "betweenness", "closeness",

"expectedInfluence"))

dev.off()

pdf("1601_centrality_stability_no_aut.pdf")

plot(Centrality_stability_no_aut,

statistics = c("strength", "betweenness", "closeness",

"expectedInfluence"))

dev.off()

### Centrality stability coefficient

cs1 <- corStability(Centrality_stability_aut)

cs2 <- corStability(Centrality_stability_no_aut)

## run jointly estimated network using estimate group network

## and ebic estimation. This takes some time.

EGN_ebic <- EstimateGroupNetwork(list(anxdep_aut, anxdep_no_aut),

method="InformationCriterion",

strategy="sequential",

criterion="ebic",

simplifyOutput = FALSE,

seed=756, ncores=8,

covfun = cor_auto)

## inspect networks

EGN_ebic$network[[1]]

colSums(EGN_ebic$network[[1]] !=0) ## total=160

EGN_ebic$network[[2]]

colSums(EGN_ebic$network[[2]] !=0)## total=162

## visualize jointly estimated networks

## need to check here graph maximum for each graph and include

## again need to check this to reflect intended scale totals

laYout=averageLayout(aut_plot_ebic, noaut_plot_ebic, repulsion=0.8)

## create curvature of networks

## these need to be adjusted for shared data

curve.aut<-c(7, 15, 35, 72)

curve.a<-rep(0, 80)

curve.a[curve.aut]<-c(-0.6, -0.6, 2.1, 0.2)

curve.noaut<-c(4, 6, 7, 13, 20, 19, 59, 73)

curve.n<-rep(0, 81)

curve.n[curve.noaut]<-c(-0.5, -0.8, 3.3, -0.6, 2.4, -1.0, -0.15, 0.4)

pdf("1601_groupnetwork_ebic_trans1.pdf", width=14, height=8)

par(mfrow=c(1,2))

aut_plot_ebic <- qgraph(EGN_ebic$network[[1]],

title="Autistic people",

layout="spring", labels = laBels_group,

esize=25, curve=curve.a,

theme="colorblind", groups=groUps,

border.width=3, vsize=12,

border.color='#555555', legend=FALSE,

maximum=0.95, minimum=0.08, cut=0.08)

noaut_plot_ebic <- qgraph(EGN_ebic$network[[2]],

title="Non-autistic people",

layout=aut_plot_ebic$layout,

labels = laBels_group,

groups=groUps, curve=curve.n,

theme="colorblind", esize=25,

border.width=3, vsize=12,

border.color='#555555', legend=FALSE,

maximum=0.95, cut=0.08,

minimum=0.08)

dev.off()

## check absolute edge differences

input<-abs(getWmat(EGN_ebic$network[[1]])-

(getWmat(EGN_ebic$network[[2]])))

input

which(input==max(input), arr.ind=TRUE)

which(input>=tail(sort(input), n=10)[1], arr.ind=TRUE)

## check centrality

## expected influence

pdf("1601_compare_EI_trans.pdf", height=5, width=4)

centralityPlot(list(autistic=aut_plot_ebic,

nonautistic=noaut_plot_ebic),

include="ExpectedInfluence",

orderBy = "ExpectedInfluence")

dev.off()

## now run with raw scores and see how it looks as in

## Burger 2020, p.20 footnote

pdf("1601_compare_EI_trans_raw.pdf", height=5, width=4)

centralityPlot(list(autistic=aut_plot_ebic,

nonautistic=noaut_plot_ebic),

include="ExpectedInfluence",

orderBy = "ExpectedInfluence", scale=c("raw"))

dev.off()

## check strength, closeness and betweenness

pdf("1601_compare_others.pdf", height = 5, width=6)

centralityPlot(list(autistic=aut_plot_ebic, notautistic=noaut_plot_ebic),

include=c("Strength",

"Betweenness", "Closeness"),

orderBy = "Strength")

dev.off()

## identify 75th centile expected influence nodes

exp_aut1<-expectedInf(aut_plot_ebic, step = 1)

exp_noaut1<-expectedInf(noaut_plot_ebic, step = 1)

exp_inf_aut1 <- exp_aut1$step1

top_exp_inf_aut1 <- names(exp_inf_aut1[exp_inf_aut1>quantile

(exp_inf_aut1, probs=0.75,

na.rm=TRUE)])

top_exp_inf_aut1

exp_inf_noaut1 <- exp_noaut1$step1

top_exp_inf_noaut1 <- names(exp_inf_noaut1[exp_inf_noaut1>quantile

(exp_inf_noaut1, probs=0.75,

na.rm=TRUE)])

top_exp_inf_noaut1

## run network comparison test

## first check how similar pearson and polychoric correlations are as NCT

## only uses pearson

## we already have missing cases excluded pairwise

## check correlation of pearson, versus spearman correlation

c1 <- cor(anxdep_aut)

c1b <- cor_auto(anxdep_no_aut)

cor(c1[lower.tri(c1)], c1b[lower.tri(c1b)], method="spearman") #0.87

c2 <- cor(anxdep_aut)

c2b <- cor_auto(anxdep_no_aut)

cor(c2[lower.tri(c2)], c2b[lower.tri(c2b)], method="spearman") #0.87

## since transforming the data these now correlate 1

## then test absolute differences in edge weights and centrality

## check absolute edge differences

input<-abs(getWmat(EGN_ebic$network[[1]])-

(getWmat(EGN_ebic$network[[2]])))

which(input==max(input), arr.ind=TRUE)

which(input>=tail(sort(input), n=10)[1], arr.ind=TRUE)

## plot edge differences

pdf("1601_differences.pdf", width=6, height=6)

plot.diff<-qgraph(input, title="Largest absolute differences",

layout=aut_plot_ebic$layout,

labels = laBels_group,

groups=groUps,

theme="colorblind", esize=25,

border.width=3, vsize=12,

border.color='#555555', legend=FALSE,

maximum=0.14, cut=0.07,

minimum=0.00)

dev.off()

## check absolute difference in node centrality

expinfdiff<-exp_aut1$step1-exp_noaut1$step1

expinfdiff

## nodes with greatest real difference in expected influence

## difference >+-0.1 - outsider -0.18

## close -0.14, rid -0.1, tired -0.14, depressed 0.13, dead 0.1

## first run NCT on largest absolute edges and nodes

## then run NCT on largest abs edge differences

set.seed(159)

NCTres1 <- NCT(anxdep_aut, anxdep_no_aut,

it=5000, binary.data=FALSE,

test.edges=TRUE,

edges=list(c(5,3), c(10,4),

c(12,9), c(10,14), c(13,11)),

progressbar=TRUE, test.centrality = TRUE,

centrality="expectedInfluence",

nodes=c(4,6,10,12,13,14))

## retrieve measurements

NCTres1$glstrinv.real ## 0.38 difference in global strength between networks

NCTres1$glstrinv.sep ## 9.64 autistic group; 10.02 non-autistic group ## gives strength for each group

NCTres1$glstrinv.pval

NCTres1$nwinv.real ## the value of the maximum difference M in any of the edge weights

NCTres1$nwinv.pval

NCTres1$einv.real ## value of difference in edge weight of the observed network

NCTres1$einv.pvals

NCTres1$einv.pvals[which(NCTres1$einv.pvals[,3]<0.05),] ## edges that differ significantly

## retrieve differences in centrality estimates

NCTres1$diffcen.real

NCTres1$diffcen.pval

NCTres1$diffcen.sep

## then run NCT on all edges and nodes

set.seed(17)

NCTres2 <- NCT(anxdep_aut, anxdep_no_aut,

it=5000, binary.data=FALSE,

test.edges=TRUE, edges='all',

progressbar=TRUE, test.centrality = TRUE,

centrality="expectedInfluence",

nodes='all')

## retrieve measurements

NCTres2$glstrinv.real ## 0.38 difference in global strength between networks

NCTres2$glstrinv.sep ## 9.64 autistic group; 10.02 non-autistic group ## gives strength for each group

NCTres2$glstrinv.pval

NCTres2$nwinv.real ## the value of the maximum difference M in any of the edge weights

NCTres2$nwinv.pval

NCTres2$einv.real ## value of difference in edge weight of the observed network

NCTres2$einv.pvals

NCTres2$einv.pvals[which(NCTres2$einv.pvals[,3]<0.05),] ## edges that differ significantly
