# Supplementary material for: The role of anxiety and depression in suicidal thoughts for autistic and non‐autistic people: A theory‐driven network analysis
Source: Suicide Life Threat Behav. 2023 Mar 28;53(3):426–42. doi: 10.1111/sltb.12954 (PMC10947106; doi:10.1111/sltb.12954)
Supplement: Supplementary file 2 — Figure S1 [file SLTB-53-426-s002.docx]

Supplementary Information 2

**Supplementary Figure 1a: Output of the *networktree* analysis**

This shows that the data splits primarily on autism diagnosis with data from non-autistic people showing a different pattern to autistic people and those seeking or not seeking diagnosis. Only the non-autistic data split according to gender.

**Supplementary Figure 1b: Unstandardized relative expected influence of nodes within autistic and non-autistic networks**
