# Supplementary material for: The role of anxiety and depression in suicidal thoughts for autistic and non‐autistic people: A theory‐driven network analysis
Source: Suicide Life Threat Behav. 2023 Mar 28;53(3):426–42. doi: 10.1111/sltb.12954 (PMC10947106; doi:10.1111/sltb.12954)
Supplement: Supplementary file 3 — Figures S2–S7 [file SLTB-53-426-s003.docx]

**Supplementary Information 3**


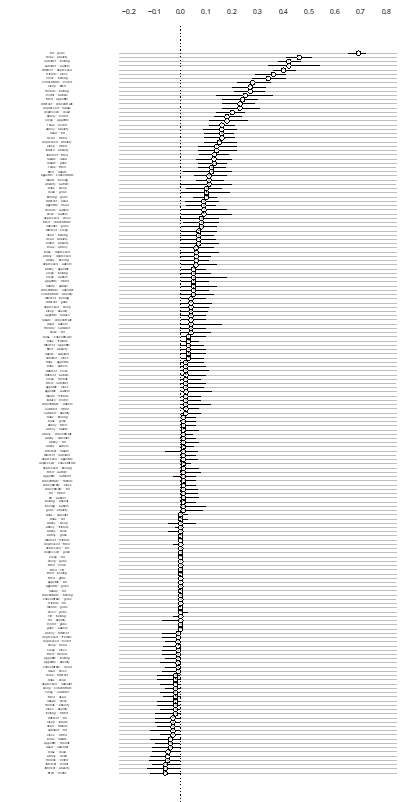


**Supplementary Figure 2: Accuracy of edge-weights and their bootstrapped confidence intervals in whole sample network**


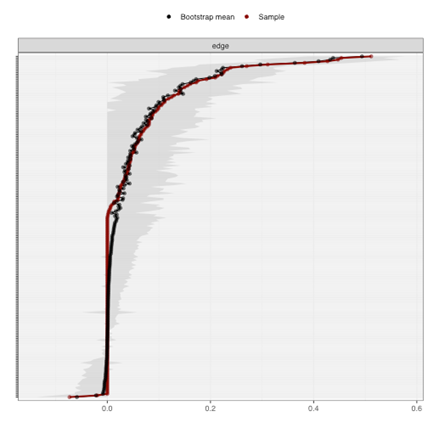


**Supplementary Figure 2: Accuracy of edge-weights and their bootstrapped confidence intervals for non-autistic people**


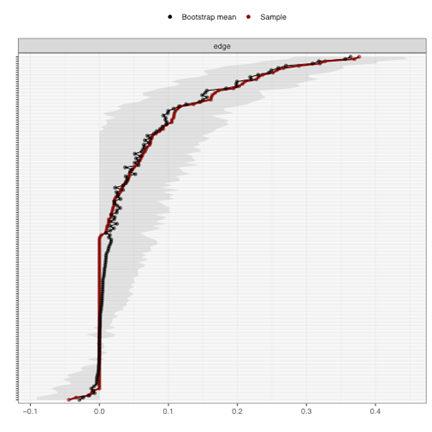


**Supplementary Figure 3: Accuracy of edge-weights and their bootstrapped confidence intervals for autistic people**

In Supplementary Figures 2 and 3, the red line represents the sample edge values, the black line represents the bootstrapped sample, and the grey area represents the bootstrapped confidence interval. The sample edge values fall within the bootstrapped confidence interval suggesting accurate edge weight estimations. The y-axis represents each edge. The edge labels are removed to avoid cluttering.

**Supplementary 5: Edge weight difference tests**


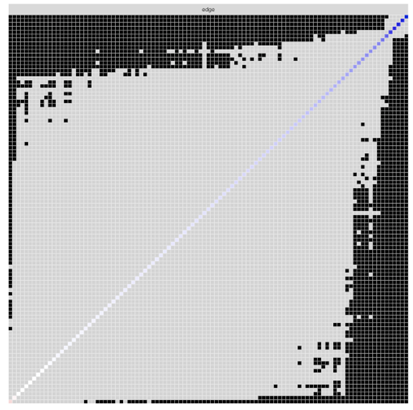


**Supplementary Figure 4: Edge weight difference test in non-autistic people**


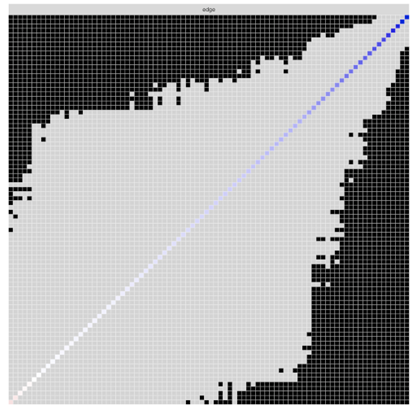


**Supplementary Figure 5: Edge weight difference test in autistic people**

In Supplementary Figures 4 and 5, grey boxes represent edges that do not differ significantly from one another whilst black boxes represent edges that differ significantly from each other. Where black boxes are numerous, this suggests that many edges differ from each other suggesting that the network’s stability can be interpreted as high. Edge labels are removed to avoid cluttering.

**Supplementary 6: Stability of centrality estimates**


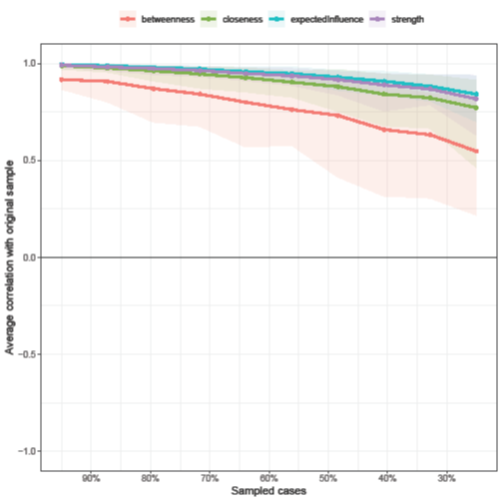


**Supplementary Figure 6: Stability of centrality indices for autistic people**

The CS-coefficient indicated that the expected influence stability was 0.75 which suggests that Expected Influence is stable in the autistic group.


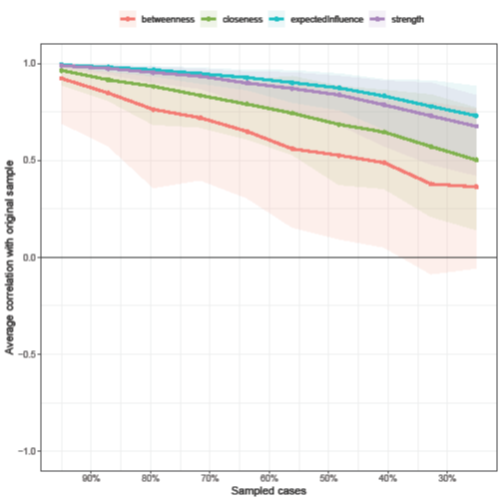


**Figure 7: Stability of centrality indices in the non-autistic group**

The CS-coefficient indicated that the expected influence stability was 0.52 which suggests that Expected Influence is stable in the non-autistic group.
